# Editorial

**Published:** 1854-06

**Authors:** 


### THE MEDICAL EXAMINER.

### PHILADELPHIA, JUNE, 1854.

A full report of the Proceedings of the late meeting of the American Medical Association at St. Louis, will be found in this number of the Examiner. The meeting, we learn, was in every respect agreeable and satisfactory; and our friends from Philadelphia, who were present, express, in the warmest terms, their sense of the overflowing kindness and hospitality, public and private, which was lavished upon them by the profession and citizens of St. Louis.

It will be seen from the Proceedings, that the Presidency of the Association was conferred upon DR. CHARLES A. POPE, of St. Louis, a high but merited compliment to a gentleman, who, though young in years, has won an enviable personal and professional position. Dr. Pope discharged the duties of the Chair with eminent courtesy dignity, and propriety.

In the number and interest of the *Reports,* we have no doubt that the volume of Transactions for the present year will be found in no way inferior to any of its predecessors. We regret, however, to perceive that a practice has grown up of holding back the presentation of Reports from the meeting of the Association, under the expectation that the authors will be permitted to complete them at their leisure, and afterwards to hand them to the Committee of Publication. This indulgence, though under certain circumstances no doubt occassionally proper, may lapse into an abuse, and it appears to us that it would perhaps be best in no case to allow papers to go into the volume of Transactions, which are not actually presented to the Association. We should prefer, too, as a general rule, to hear all Reports read in extenso, and not condensed into *abstracts*, which in some cases are so brief as scarcely to convey an idea of the bearing of the papers. Can the time of the Association be better employed than in hearing these Reports? And, apart from the interest and profit which they afford, is it not proper that the Association should be made acquainted with the matter which it is about to send forth as a portion of its Transactions?

Under the present system of Committees on Specialties, five years are, we believe, allowed a Committee for the completion of a Report. A considerable number of the Committees very properly asked and obtained extension of time, and were continued. There are certain Committees, however, Reports from which are obviously expected at each session; and some of these were not forthcoming. We allude to the point, because we think that hereafter Chairmen of Committees on topics of immediate and passing interest, should either prepare Reports or make a timely transfer of their duties.

The amendments to the Constitution on the subject of Representation, were not called up. What their fate would have been, and what was the temper of the Association in regard to them, must be left to speculation, as the matter was not in any way introduced. The want of concert of action on the part of the friends of reform, and of arrangement to bring the subject forward, evince, however, a decline of interest in the question, and the absence of any strong desire to disturb the present organization of the Association.

The list of Committees shows a change in the constitution of the Committee of Publication. Since the foundation of the Association, a majority of this Committee has been selected from the profession of Philadelphia, with the understanding that the Transactions would be issued in this city. A majority of the Committee is now, however, taken from the profession of New York, with the understanding that the publication of the Transactions is transferred to the latter city. This change was not made without considerable discussion. It was contended, on the one hand, that it was better to retain the experience and tried ability of the gentlemen who have so long superintended the publication of the Transactions in Philadelphia, and that the facilities for their early and creditable issue in this city, were not surpassed in other places. On the other hand, a strong feeling was evinced in favor of a distribution of the honors and labors of the Association, and this feeling finally prevailed. The discussion of the proposed removal was animated, but conducted with entire courtesy and good feeling. And, in referring to the topic, we beg to say, that we bow most cheerfully to the expressed decision of the Association, in the exercise of its undoubted right upon the subject, and that we have every reason to anticipate that the zeal and industry of our friends in New York will do early and ample justice to the papers committed to their hands.

A resolution was passed by the Association, that the majority of the Committee of Publication shall hereafter be taken from the profession of the city in which the Association may meet. The principle of rotation in all its offices appears to be thus established as the settled policy of the Association. A majority of the Publication Committee will in accordance with this resolution, next year be selected from Philadelphia, and thereafter annually changed with the place of meeting.

Another resolution, passed with reference to the Publication of the Transactions, prohibits the issue, in separate form, of any paper included in the volume of the Transactions. Objection was especially made to the use of types or plates prepared at the expense of the Association, by a publishing house, for a nominal consideration; and it was argued, with justice, certainly, that such separate issue is a material interference with the sale of the Transactions.

By the selection of Philadelphia for the next annual session of the Association, the profession of Philadelphia feels greatly complimented and gratified. We hope to be able to make the meeting pleasant to our friends, whom we trust to see from every section of the Union, and we beg to assure them that we are not unmindful of the noble courtesy which has been received, for so long a series of years, by the profession of Philadelphia from her sister cities.

---

In the July number we shall publish an article by Dr. Brinton, upon a new way of preserving anatomical specimens, animal substances, &c., from decomposition. The perfectly successful preservation of the articles exhibited to us, induces us to think very highly of its value.

